# High-dose chemotherapy followed by whole lung irradiation in pulmonary relapse Ewing’s sarcomas: a retrospective multicenter study

**DOI:** 10.1259/bjr.20220212

**Published:** 2022-07-12

**Authors:** Loig Duverge, Charlotte Demoor-Goldschmidt, Anne Laprie, Mathilde Cervellera, Marie Pierre Castex, Nadège Corradini, Perrine Marec-Berard, Line Claude

**Affiliations:** 1 Radiation Oncology Department, Centre Eugène Marquis, Avenue Flandres Dunkerque, Rennes, France; 2 Pediatric Oncology, Institut de cancérologie de l’Ouest-Paul Papin, Angers, France; 3 Radiation Oncology Department, IUCT Oncopole, Toulouse, France; 4 Radiation Oncology Department, Hôpital privé Jean Mermoz, Lyon, France; 5 Department of Pediatric Oncology, children’s hospital, Toulouse, France; 6 Department of Pediatric Oncology IHOPE, Centre Léon Bérard, Lyon, France; 7 Radiation Oncology Department, Centre Léon Bérard, Lyon, France

## Abstract

**Objective:**

Regarding the efficiency of Bu-Mel-based high-dose-chemotherapy (Bu-Mel-HDCT) and whole lung irradiation (WLI), the objective was to evaluate the efficiency and safety of this salvage sequence in Ewing sarcoma (ES) lung relapses.

**Methods:**

All eligible pediatric ES patients (1991–2020) identified in SFCE departments were retrospectively reviewed. Seven patients were (1) diagnosed with a pulmonary relapse, isolated or not, (2) naïve from both HCDT and WLI (3) treated by the salvage sequence of conventional chemotherapy, Bu-Mel-HDCT and WLI. The main endpoint was OS evaluation. WLI toxicities were scored using CTC-V5.

**Results:**

With a 13 years median follow-up (FU), 5/7 patients are alive and in complete remission. 10y-EFS is 71.4%. Three patients experienced transitory radio-induced pneumopathy (RIP). A patient developed RIP (gr.3) and finally progressive lung fibrosis leading to death.

**Conclusion:**

This study reports seven ES patients treated for lung metastatic relapses, using an aggressive strategy, with favorable survival long-term results which should be balanced with the risk of lung toxicity.

**Advances in knowledge:**

The approach of surgery, Bu-Mel HDCT followed by WLI can be discussed in selected ES patients with lung relapse, naive from HDCT or WLI, providing an optimal chemosensitivity. A special vigilance is necessary regarding the incidence rate of lung toxicity which can be mitigated by limiting the radiotherapy dose, and observing optimal timing of radiotherapy after HDCT.

## Introduction

The prognosis of Ewing’s sarcoma (ES) has been improved by multimodal treatment with a 5 year event-free survival (EFS) of 65–72% and 21–28% for localized and metastatic diseases respectively.^1–3^

In case of relapse, the prognosis of ES is poor with 5-year overall survival (OS) about 10–20%.^1,4^ Multiple metastatic ES relapses lead to a 5-year EFS about 5–12%.^1,2,5^ The prognosis seems better in case of isolated local relapse (<10% of cases), or only pulmonary relapse, with a 5-year EFS about 20–34%.^2,5^ Late relapses (>24 months) also appear to be associated with a better prognosis with a 5-year EFS about 29–45%.^1,2,4^

No standard treatment is clearly defined in ES relapses, but the most common attitude remains to carry out a second-line palliative chemotherapy based on Temodal/Irinotecan, Topotecan/Cyclophosphamide or Carboplatin-based chemotherapy.^3,6,7^ The results of the Phase II/III study rEECur (https://www.clinicaltrialsregister.eu/ctr-search/trial/2014-000259-99/BE#A) are awaited to determine the best chemotherapy strategy. Tyrosine kinase inhibitors may represent a new therapeutic option.^8^

In selected favorable situations (late relapses, isolated local recurrence and/or only lung relapses) aggressive strategies have been reported, including high-dose chemotherapy (HDCT)^2,9^ and whole lung irradiation (WLI).^10^ A retrospective study (*N* = 73 patients naive from HDCT) showed a benefit from a second-line treatment based on Busulfan Melphalan based HDCT (Bu-Mel-HDCT) versus conventional chemotherapy with a 2-year EFS of 47 *vs* 10% in favor of Bu-Mel-HDCT.^9^ WLI improves first-line recurrence-free survival for pleural or pulmonary metastatic ES with a progression-free survival of 40% at 5 years in the event of bipulmonary irradiation *vs* 19% without this irradiation.^11^ For these first-line patients with lung metastasis, the combination of conventional chemotherapy (vincristine, dactinomycin and ifosfamide) and WLI remains the reference treatment *vs* chemotherapy with Bu-Mel according to Euro-Ewing99 results.^12^ The efficacy of WLI in case of pulmonary metastatic recurrence is unknown but may have an effect similar to that shown in patients on the first-line treatment of relapse.^10^

The toxicity of WLI and conventional polychemotherapy combination is described as acceptable in the literature.^13^ However, the synergistic effect of the combination of radiotherapy and Bu-Mel-HDCT has been described. Rare but severe toxicities (myelitis, digestive, brain necrosis…) have been reported.^14,15^

Regarding the efficiency of both Bu-Mel-HDCT and WLI, the question whether this combination may be efficient and safe in case of lung relapse ES is still under debate and not yet evaluated.

The main objective of this study is to evaluate both efficacy and toxicity of this therapeutic sequence in ES patients with pulmonary relapse.

## Methods

All pediatric ES patients with the following criteria between 1991 and 2020 were identified in all the pediatric/adolescents and young adults (AJA) oncology departments of Société Française de lutte contre le Cancer et leucémies de l’Enfant et de l’adolescent (SFCE) centers and were retrospectively reviewed:

–Patients diagnosed with a pulmonary relapse, isolated or not, naïve from both HCDT and WLI during the first-line treatment
–Treated by the salvage sequence: conventional salvage chemotherapy, Bu-Mel-HDCT followed by WLI

Consent was obtained from all alive individual participants (or parents if minor patients) included in the study (MR004 formula: V3.1 23/01/2020).

The main end point was OS evaluation. Secondary end points included EFS and WLI toxicity evaluations. OS was calculated from the diagnosis of relapse to the date of death from any cause.

EFS was calculated from the diagnosis of relapse to the date of the first recurrence or death.

Acute and late WLI toxicities delivered after Bu-Mel-HDCT were scored according to the CTC-V5 classification.

## Results

Seven patients met the inclusion criteria. Median age at relapse was 15 years (range 8–20 years). Four patients had only lung metastasis at the time of relapse, while three others had lung and other metastatic sites. Median time of relapse following the initial diagnosis was 40 months (range 13–68 months).

**Figure 1.**
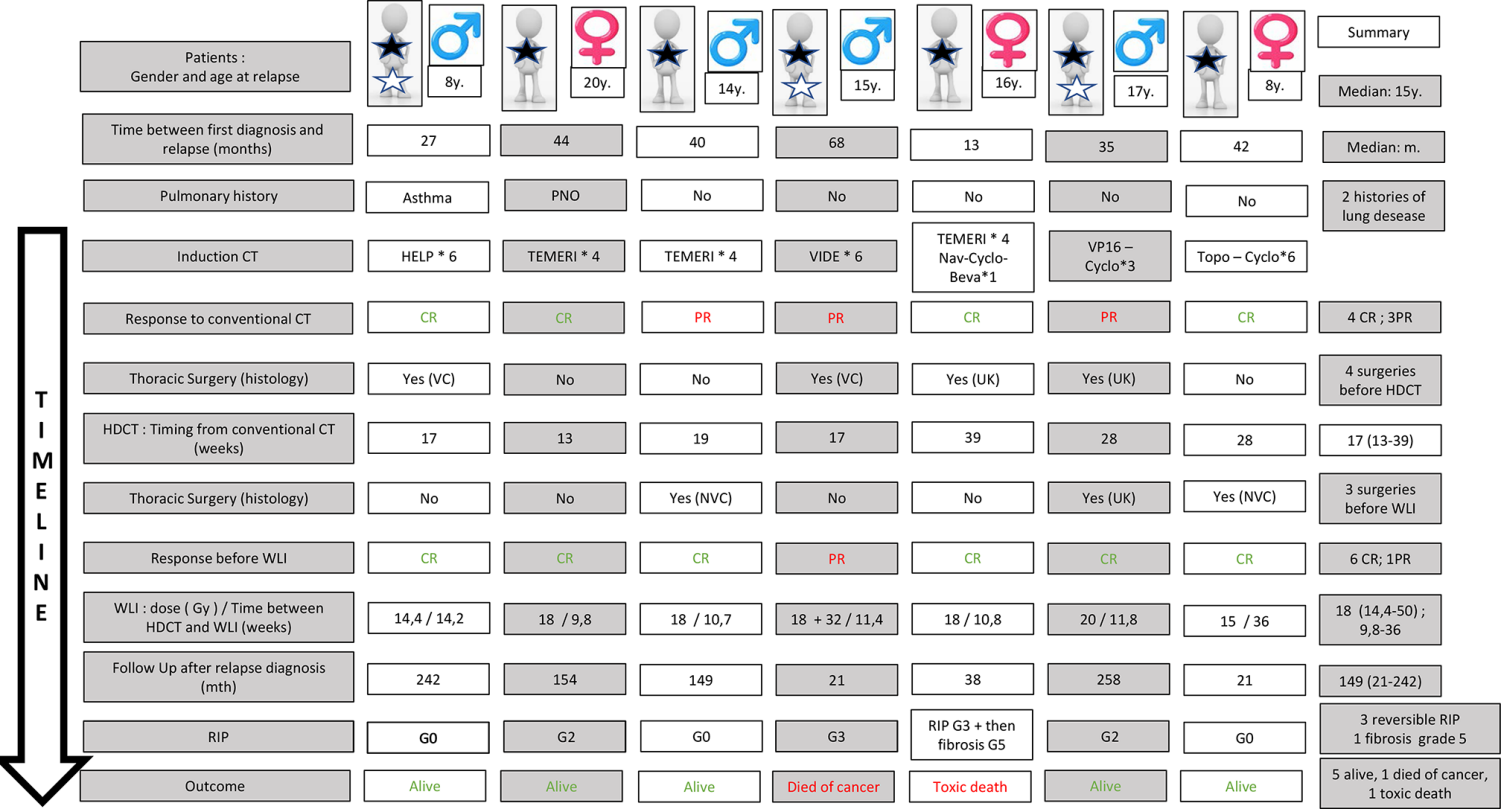
Presents salvage treatment details. Black stars represent lung metastasis at the time of relapse. White stars represent other metastatic sites (bone, bone marrow) at the time of relapse. Successive treatments are presented chronologically for each patient. Thoracic surgery was done either before or after HDCT (or both in Patient 6). If surgery was performed, “yes” appears in the box and the histological response was thus put in brackets if the information was known. If surgery was not performed, “no” appears in the box. y., years; CT, chemotherapy; VIDE, Vincristine-Ifosfamide-Doxorubicin-Etoposide; HELP, Ifosfamide-Vindesine-Cisplatin; TEMIRI, Temozolomide-Irinotecan; Nav, Navelbine; Beva, Bevacizumab; Topo, Topotecan; Cyclo, Cyclophosphamide; HDCT, high dose chemotherapy; WLI, whole lung irradiation; Gy, Grays; CR, Complete Response; PR, Partial Response; VC, viable cell; NVC, no viable cell; UK, Unknown; PNO, pneumothora ; RIP, Radiation-induced Pneumopathy; mth, months; G, grading of adverse event according to CTCAE v. 5.

Figure 1 presents salvage treatment details.

Briefly, all patients received first three to six cycles of conventional second-line chemotherapy. A complete (*n* = 4) or partial (*n* = 3) response was achieved using RECIST criteria. They were thus all considered as chemosensitive ES patients. The conventional chemotherapy was followed by Bu-Mel-HDCT (Busulfan (600 mg/m^2^), Melphalan (140 mg/m^2^)) and autologous cell stem transplantation (ASCT). Lung metastasectomy was performed in 6/7 patients either before Bu-Mel-HDCT (*N* = 3), just before WLI (*N* = 2) or both (*N* = 1). As a consequence, 6/7 patients had achieved complete lung remission before WLI. WLI was delivered using conformal radiation (*N* = 6) or Tomotherapy^®^ (*N* = 1). Median total dose was 18 Gy (14.4–20) in 1.5–2 Gy/fraction. All patients over 14 years received 18–20 Gy while the younger patients received 14.4–15 Gy. Median time between Bu-Mel-HDCT and the beginning of WLI was 11.4 weeks (10–52).

With a median follow-up of 16.3 years (range 5.7–24.6) after the initial diagnosis and 13.0 years (range 1.75–21.7) after the relapse, 5/7 patients are alive and in complete response. 10-year OS was 71.4%. The 3-year EFS and 10-year EFS were 85.7 and 71.4% respectively.

Table 1 presents the results. Two patients died: one died of metastatic lung progression 1.75 years after the end of WLI.

**Table 1.** Parameters of relapse, toxicity of treatment relapse and survivals in all the patients

Patient	Site of relapse	Pulmonary toxicity (time interval after WLI in months)	Treatment (length)	Reversibility of symptoms	Last pulmonary function test (time interval after WLI in months)	Survival since diagnosis (months)	Survival since relapse (months)	Death / cause of death
1	Metastatic (right lung, mediastinum, bone marrow)	Asymptomatic mild restrictive syndrome (262)	None	No symptom	FVC : 64% FEV1: 68% (242)	297	249	Alive
2	Metastatic (lungs)	RIP G2 (3)	None	Yes	FVC : 62% FEV1: 57% DLCO: 81% (154)	203	159	Alive
3	Metastatic (lungs)	None	None	No symptom	No data available	196	156	Alive
4	Local only and then metastatic (lung and mediastinum)	RIP G3 (10)	Antibiotherapy Corticotherapy (6 weeks)	Yes	No data available	113	27	Yes / Pulmonary relapse
5	Metastatic (lungs)	RIP G3 (3) Pneumocystosis (4) Carcinologic PNO (12) G5 Pulmonary fibrosis(33)	Long-term corticotherapy (2 years) Oxygenotherapy	No	FEV1: 0,48L (15%) FVC: 0,5L (14%) FEV1/VC:94% (26)	64	51	Yes / Toxic death
6	Metastatic (lungs, left upper limb and subcutaneous)	RIP G2 (18)	Corticotherapy (12 months)	Yes	FEV1/VC: 99% FEV1:77% DLCO:60% CPT: 76% (252)	295	260	Alive
7	Metastatic (lungs)	None	None	No symptom	No data available	76	21	Alive

RIP, radio-induced pneumopathy; WLI, whole lung irradiation.

The other patient died from lung treatment toxicity 2.8 years after the end of WLI (described below).

Acute WLI toxicity after Bu-Mel-HDCT was low. One chest radiodermatitis (gr.1) was reported.

Regarding middle/long-term toxicity, four patients experienced RIP (two gr.2 and two gr.3). They presented cough/dyspnea associated with interstitial syndrome on CT scan. All had been previously treated with lung surgery. Median time between surgery and WLI was 3.9 months (range 1.3–6.5 months). RIP appeared with a median time of 6.2 months following WLI (range 1.6–16.1 months). In three patients on four, RIP was reversible within few weeks using steroids (*N* = 2) or without any treatment (*N* = 1). In the fourth patient, RIP gr.3 appeared 3 months after WLI. A wedge resection had been performed 3.9 months before WLI. RIP was associated with pneumocystis infection and required transitory oxygenotherapy in the following months. The RIP led to a severe progressive lung fibrosis, complicated by several pneumothoraxes. The patient finally died from acute respiratory distress syndrome 2.8 years after WLI without sign of disease progression and 2.6 years after the beginning of RIP.

Two patients experienced late thyroid toxicity. One patient developed hypothyroidism 2.8 years after WLI. One patient had a thyroid cancer 7.7 years after WLI. No other late toxicity was reported.

## Discussion

With a long median follow-up, this series reports 7 ES patients treated for lung metastatic relapse using an aggressive strategy including a second-line chemotherapy, lung surgery (6/7 patients), and Bu-Mel-HDCT followed by WLI.

A strategy combining Bu-Mel-HDCT/WLI has already been retrospectively evaluated with encouraging EFS results on 102 patients in first-line metastatic ES. Lung toxicity was acceptable after a lower WLI dose (12–15 Gy in 10 fractions).^16^ To our knowledge, our study is the first one reporting the use of a salvage treatment using Bu-Mel-HDCT/WLI in the situation of relapse.

The survival results (10-y.EFS 71.4%) compare favorably with the literature data which reports 5-year EFS between 20 and 34%^3,6^ in the situation of lung relapse. In addition, 3/7 patients had a non-isolated lung relapse (bone marrow, bone and/or nodes involvement): 2/3 are free of disease with a follow-up longer than 10 years despite a very poor prognosis, with 5-year EFS reported below 15% in the literature.^1,2,5^

Of note, some favorable prognostic factors may explain the good outcome of the cohort^1^: the chemosensitivity at relapse was good in all patients. Four and three patients on seven were in complete remission or partial remission respectively after the salvage induction chemotherapy, before Bu-Mel HDCT decision^2^ all patients were in complete remission of the lung metastasis before WLI, either with chemotherapy alone (*N* = 1), or after chemotherapy and lung surgery (*N* = 6). As a consequence, WLI was performed in minimal disease situation which may explain a better efficiency.

These good results in term of outcome must be balanced by the risk of lung toxicity. Four patients on seven developed RIP. Three of them had a Grade 2 or 3 transitory RIP, which required steroids in two patients, with reversible symptoms. The fourth patient died of late lung fibrosis despite long-term steroids and oxygen therapy after a RIP (Grade 3) complicated by pneumocystosis infection and then several pneumothoraxes.

The synergistic effect of the combination of WLI and Bu-Mel-HDCT has already been reported, with severe digestive radiation toxicity and myelitis after radiation doses usually non-toxic in the absence of Busulfan.^14^ ^15^ However, a better selection of patients and strict radiation dose limitations on critical organs after Bu-Mel-HDCT probably limit the risk. A recent literature review, showed especially that the risk of lung toxicity is reduced after WLI dose <15  Gy and a time-interval between HDCT/WLI > 60 days.^17^

Despite bias inherent to a retrospective study and a limited number of patients due to a rare situation, we report that the combination of Bu-Mel-HDCT and WLI was probably a curative strategy in 4/7 patients, alive and in CR more than 13 years after relapse. One additional patient is also in CR with a more limited follow-up. These patients are known to have a poor prognosis. Our results suggest that this aggressive strategy may be discussed in selected patients naive from HDCT or WLI in first-line, with late lung relapse, providing an optimal chemosensitivity to a second-line conventional chemotherapy (complete/very good partial lung response). In case of residual lung metastasis, despite no clear published data, surgery should be discussed to achieve CR before WLI. The lung toxicity was however significant. The four patients who developed lung toxicity received a total WLI dose of 18 Gy or more. The recent literature suggests a lower prescribed dose may be safer. In this rare situation, WLI dose may probably be limited to 15 Gy in 10 fractions and the interval between Bu-Mel-HDCT and WLI should be longer than 60 days.^16,17^

## Conclusions

This retrospective study included only a small number of patients due to the rare context of lung relapse of ES. Regarding the reported efficiency, an aggressive strategy including Bu-Mel HDCT and WLI could be discussed in very selected ES patients with a late limited lung relapse, naive from HDCT or WLI in first-line treatment, providing an optimal chemosensitivity to a second-line conventional chemotherapy. However, regarding the high lung complication risk, only patients with normal function tests should be discussed for this combination, and both the timing of the WLI after Bu-Mel HDCT and the limited radiation dose should be respected.
